# Patient-initiated cardiovascular monitoring with commercially available devices: How useful is it in a cardiology outpatient setting? Mixed methods, observational study

**DOI:** 10.1186/s12872-022-02860-x

**Published:** 2022-09-29

**Authors:** Christine A’Court, Wilfred Jenkins, Claire Reidy, Chrysanthi Papoutsi

**Affiliations:** Nuffield Department of Primary Care Health Sciences, University of Oxford, Oxford, UK

**Keywords:** Remote monitoring, Digital health, Self-monitoring, Arrhythmias, Atrial fibrillation

## Abstract

**Background:**

The availability, affordability and utilisation of commercially available self-monitoring devices is increasing, but their impact on routine clinical decision-making remains little explored. We sought to examine how patient-generated cardiovascular data influenced clinical evaluation in UK cardiology outpatient clinics and to understand clinical attitudes and experiences with using data from commercially available self-monitoring devices.

**Methods:**

Mixed methods study combining: a) quantitative and qualitative content analysis of 1373 community cardiology clinic letters, recording consultations between January–September 2020 including periods with different Covid-19 related restrictions, and b) semi-structured qualitative interviews and group discussions with 20 cardiology-affiliated clinicians at the same NHS Trust.

**Results:**

Patient-generated cardiovascular data were described in 185/1373 (13.5%) clinic letters overall, with the proportion doubling following onset of the first Covid-19 lockdown in England, from 8.3% to 16.6% (p < 0.001). In 127/185 (69%) cases self-monitored data were found to: provide or facilitate cardiac diagnoses (34/127); assist management of previously diagnosed cardiac conditions (55/127); be deployed for cardiovascular prevention (16/127); or be recommended for heart rhythm evaluation (10/127). In 58/185 (31%) cases clinicians did not put the self-monitored data to any evident use and in 12/185 (6.5%) cases patient-generated data prompted an unnecessary referral. In interviews and discussions, clinicians expressed mixed views on patient-generated data but foresaw a need to embrace and plan for this information flow, and proactively address challenges with integration into traditional care pathways.

**Conclusions:**

This study suggests patient-generated data are being used for clinical decision-making in ad hoc and opportunistic ways. Given shifts towards remote monitoring in clinical care, accelerated by the pandemic, there is a need to consider how best to incorporate patient-generated data in clinical processes, introduce relevant training, pathways and governance frameworks, and manage associated risks.

## Background

The Covid-19 pandemic contributed to fragmentation in service provision including in cardiovascular care, where there was a drop in emergency presentations to secondary care, restrictions on investigations and interventions due to infection control and reduced capacity, and suspension of elective procedures in some outpatient and community specialist nursing services in the UK [1]. At the same time, this disruption led to increased reliance on technology and self-monitoring in many areas of healthcare [2]. In specialist cardiovascular care, there has been a shift to remote patient monitoring, for example using data from implanted cardiac devices such as permanent pacemakers and implantable cardiac defibrillators [3]. In primary care, the deployment of home blood pressure monitors and pulse oximetry has been promoted in specific clinical settings [4].

Commercially available digital technologies, such as wearables, have long been promoted in the context of fitness and wellbeing, with worldwide sales predicted to reach 525 million by 2025 [5]. These devices, developed primarily for recreational purposes, are now increasingly adapted and promoted to fulfil healthcare and self-monitoring needs, and promise enhanced insights to cardiovascular health, potential for self-care and contribution to long-term conditions management [6]. Commercial activity trackers, smartphones and smartwatches, offering access to heart rate monitoring, propose convenience over traditional and clinically validated devices like blood pressure monitors and oximeters [6]. Heart rhythm monitoring can also be achieved with: certain models of home blood pressure monitors incorporating algorithmic interpretation of heart rate variability [6, 7], portable single lead electrocardiogram (ECG) recorders (such as KardiaMobile™), and smart watches and wrist-mounted fitness trackers by means of photoplethysmography, an optical technique detecting blood volume changes in peripheral tissues [8, 9]. Newer Apple Watch™ [10, 11] and Fitbit™ [12] models also provide single-lead ECG readings accompanied by some algorithmic interpretation.

Little is known about the clinical utility of patient-generated data using traditional and novel devices in cardiology settings and there are presently no concrete guidelines for primary care physicians or cardiologists on how to use, interpret or act on information from wearables and similar devices [5]. Existing cardiology literature on home monitoring, remote monitoring and telehealth focuses on clinical trials, for example investigating a single device, platform or initiative in selected patients, deployed in specific care pathways, often within specialist environments to assess impact on cardiovascular outcomes such as death and hospitalisation rates [13–16]. While important, these studies give little indication of opportunities and challenges when patients use commercially available monitoring devices at home and patient-generated data become introduced organically into non-experimental care environments. There is a need to understand current practice in clinical settings, to be able to learn about potential usefulness of patient-generated data for decision-making, and prepare for service implications from increased self-monitoring in the context of the pandemic and beyond.

This paper combines data from a service improvement project on the use of self-monitoring data by cardiologists in a UK NHS Trust and a qualitative research study on traditional and emerging self-monitoring devices in cardiology. We sought to: (a) quantify instances of patient-initiated home monitoring, as reported in letters by clinicians providing general community cardiology clinics, (b) characterise the contribution of patient-generated data to clinical decision-making, and c) explore clinical experiences and views of self-monitoring practices.

## Methods

### Setting

This research was conducted in the context of the Integrated Community Cardiology Service (ICCS), an outpatient cardiology service provided by ten general practitioners (GPs) with an extended role in cardiology and fully integrated with the tertiary cardiology service in a UK NHS Trust. The ICCS bridges primary and secondary care, triaging all, and seeing on average 90% of patients referred to cardiology by primary care. Recently conferred the Health Service Journal Value Award 2021: Cardiovascular Care Initiative of the Year [17], the service operates from six community-based sites across the local region and serves a patient population of over 700,000. GPs with extended role arrange co-located community-based echocardiography and ambulatory heart rhythm monitoring and have direct access to all other cardiac diagnostics provided in secondary care. The service continued almost uninterrupted throughout the Covid-19 pandemic, albeit with a temporary reduction in demand and a six-week phase of exclusive telephone or video-consultations in the first lockdown (March 2020). Since then, a mixed approach has favoured face-to face appointments but allowed remote consultations according to individual preference and self-isolation or quarantine requirements.

### Quantitative methods

To investigate the prevalence and utility of patient initiated self-monitoring data, we examined clinical letters reporting cardiology consultations by ICCS clinicians over 9 months (January–September 2020). One member of the team (WJ) reviewed letters stored on the Trust’s electronic patient record system to identify those making any mention of self-monitoring. Inclusion criteria encompassed all patients referred to the ICCS and all self-monitoring devices providing cardiovascular data, owned by patients, friends or family and mentioned in the referral proforma or ICCS clinic letters. Relevant data were then extracted on to a spreadsheet without including identifiable details (date, clinician number, patient age, device type, clinical context, and data on self-monitoring). A second member of the team and ICCS clinician (CAC) reviewed the letters mentioning self-monitoring and iteratively added contextual information to the data extracted, by matching source letters with incoming referral and follow-up letters, to enhance understanding of how self-monitoring data was used in clinical decision-making. The contribution to clinical care was assessed and categorised, a process involving discussions between the research team and progressive refining of categories for clarity and consistency.

Descriptive statistical analysis included comparisons between user age groups, references to monitoring devices before and after the first UK national lockdown (23 March 2020), and between types of remote-monitoring devices (i.e. traditional blood pressure monitors/oximeters or internet-connected devices such as smartwatches). Statistical analyses were carried out using a two-sample proportion Z-test for significant difference between the proportions of a binomial outcome between two groups.

### Qualitative methods

To extend our understanding of patient self-monitoring practices and experiences over time we recruited 20 clinicians to participate in 6 semi-structured interviews and 3 group discussions. Participants included cardiology consultants (n = 6) and trainees (n = 2), nurse practitioners (n = 2) and extended role general practitioners (n = 10). All were staff members working in secondary care at the same NHS trust; they were recruited by purposive and snowball sampling to target practitioners with roles and experience relevant to this project.

The aim of the interviews was to understand how clinicians engage with patient-generated data and the issues surrounding its use. They took place either face-to-face (where feasible) or on MS Teams due to Covid-19 restrictions, were audio-recorded (with consent) and professionally transcribed.

In the 3 group discussions we presented de-identified data from quantitative analysis to confirm, adjust or refute our initial assessment and interpretation of contribution to clinical decision-making, and to discuss implications for clinical education and service re-design. Discussions were audio-recorded and transcribed by an attending researcher (CAC), to further supplement interview data. Email discussions with attendees and other interested secondary care clinicians also contributed to the qualitative dataset.

Transcripts and field notes were read in detail by all members of the research team, with data extracted and categorised into key topic areas identified as important by interviewees and previous literature in this area, using a deductive and inductive approach. Coding was discussed between members of the team and refined during the analysis process. Qualitative data fed into the analysis and interpretation of quantitative data and vice versa, including with clinicians participating in group discussions to gain additional perspectives.

Interviews were carried out with approval from the University of Oxford’s Central University Research Ethics Committee (R68653/RE001). Review and analysis of clinical letters, and group discussions took place as part of a service improvement project.

## Results

### Quantifying self-monitoring references in clinic correspondence

We reviewed 1373 clinic letters from consultations with 10 ICCS clinicians between 1 January and 30 September 2020. Cardiovascular self- monitoring was mentioned in 185 letters (13.5%) concerning 180 unique patients (as 5 patients also had follow-up appointments during the same period). Ten individual clinicians mentioned patient self-monitoring data in 5–42 clinic letters (median 20 letters per clinician).

In 101/185 (54.6%) letters, self-monitoring was first mentioned in the originating GP’s referral, having been carried out to evaluate patient symptoms and/or produced findings that prompted referral. In the remaining 84/185 (45.4%) cases, self- monitoring information was elicited (56/84) or recommended (26/84) during the ICCS clinic consultation.

Letters referred to measurements from devices such as (see Table 1): BP monitors with or without irregular heart beat indicators (117 mentions); wearables, primarily Apple Watch™ and Fitbit™ both of which have FDA approval for their ECG, or less common digital wrist bands (43 mentions); KardiaMobile™, a NICE-endorsed and FDA-cleared handheld single lead ECG recorder with algorithmic heart rhythm interpretation (19); portable pulse oximeters displaying heart rate and oxygen saturation (13); and other technologies (4).

**Table 1:** Self-monitoring device types mentioned in community cardiology outpatient clinic letters

Device type	Mentions
BP monitor	117
Wearables (Apple Watch™, Fitbit™ etc.)	43
KardiaMobile™	19
Pulse Oximeter	13
Other (Home 12-lead ECG, stopwatch, bike monitor, iPhone pulse monitor)	4

### Overall impact of self-monitoring data on clinical decision-making or management

**Fig. 1:**
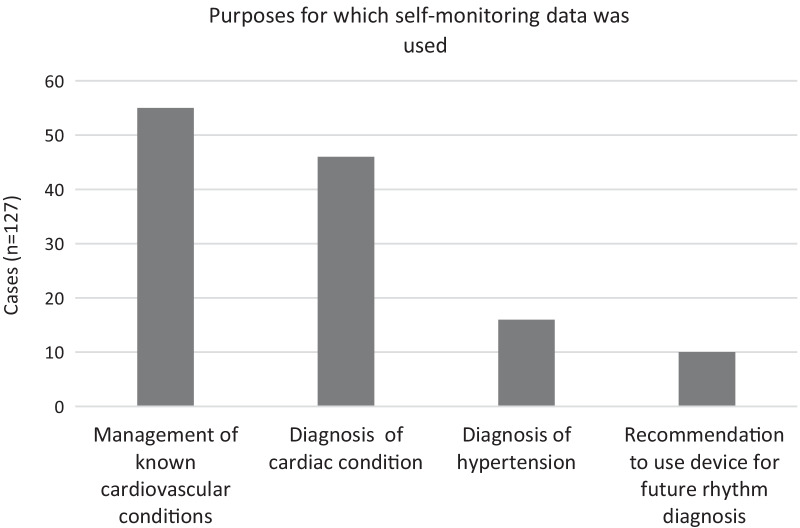
Diagram illustrating the purposes for which clinicians drew on self-monitoring data, as recorded in clinical letters

We categorised self-monitoring devices as contributing to clinical decision-making or management in 127/185 (69%) cases (Fig. 1). In a sizeable proportion (55/127), patient-supplied data assisted with managing known conditions. In 46/127 letters, patient data facilitated cardiac diagnoses. In a small minority (16/127), home BP monitoring was advised as follow up on isolated home or clinic readings indicating possible undiagnosed hypertension. Finally, in 10/127 cases a NICE-endorsed heart rhythm device was recommended to enable identification of cardiac rhythm underlying symptoms.

In 58/185 (31%) cases self-monitoring data appeared unhelpful. In 28/58 cases, patient-supplied data was recorded but appeared unrelated to presenting problems or co-morbid conditions. In 20/58 cases, data was non-specific for diagnostic purposes and even taking clinical context and symptoms into account, the clinicians were unable to either confirm or rule out important differential diagnoses. This included failure to differentiate sinus tachycardia from pathological tachyarrhythmias; or to distinguish a sinus bradycardia from frequent ectopy, bigeminy, trigeminy or significant atrioventricular conduction. Investigations and management proceeded irrespective of the data patients provided. Finally, in 10/58 cases, letters were unclear as to any influence on decision-making so assumed to be unhelpful.

### Diagnostic utility of self-monitoring data

In 46/185 (25%) cases, data was successfully used to make cardiac diagnoses. In 20/46 cases, patient devices provided clear-cut diagnostic data. All these patients presented with palpitations and around half had accompanying cardiac symptoms. In 11/20 of these cases, KardiaMobile™ devices or Apple Watch™ models provided single lead ECG traces enabling symptom-rhythm correlation equivalent to traditional ambulatory heart rhythm monitoring methods. In 2 cases, BP monitors flagged irregularity of heart rhythm indicative of AF, subsequently confirmed. In the remaining 7/20 cases, devices provided clear heart rate data, albeit without either ECG trace or algorithmic interpretation, which when combined with previous history and clinical setting facilitated diagnosis with a high degree of certainty.

In these symptomatic patients, the demonstrated rhythms were symptomatic tachyarrhythmias in 8/20 patients (4 PAF, 2 persistent AF and 2 paroxysmal SVT likely to be AVNRT). Of these 8 patients, 5 (age range 54–66 years, median 59) went on to rhythm control interventions, whilst 3 (age range 67–73 years, median 68) were managed by rate control. In 12/20 patients, symptoms coincided with benign ectopy, normal sinus rhythm, appropriate sinus tachycardia or sinus bradycardia (during vaso-vagal episodes), and patient recordings facilitated explanation and reassurance by clinicians.

In a further 14/46 cases, patient self-monitoring did not provide such clear-cut diagnostic data, but clinicians incorporated heart rate, rhythm and/or blood pressure data into their diagnostic narrative and completed management plan. Group discussions with clinicians confirmed that, although the data had no standalone diagnostic value, it was drawn on to confirm a clinical impression, sometimes to exclude alternative diagnoses and to encourage or discourage further investigation.

In 12/46 cases where device data was used for diagnostic purposes, patients were asymptomatic, and the readings had driven what could be deemed avoidable referrals. This group represents 6.5% (12/185) of the total occasions when self-monitoring was mentioned. These asymptomatic patients were referred with one or more of the following: pulse irregularity due to ectopy, bradycardias due to enhanced diurnal rhythm (in athletes), benign ectopy causing an apparent bradycardia, or exercise-related sinus tachycardias. In half of these cases Holter monitoring was instigated despite device data compatible with, and clinician expectation of an innocent explanation, evidently because the device data represented an unvalidated source of information. Although the referrals might be deemed a waste of resources, the device data was, in itself, accurate and diagnostic (of benign conditions, or normal physiology) when viewed alongside the low-risk clinical context. This small number contrasts with the perceptions of many participants in qualitative interviews (see section below) who had intense recollections of device-driven inappropriate referrals and self-monitoring findings posing no threat to the patient.

### Application of data for management of pre-existing cardiovascular conditions

In 55/185 cases, data concerning heart rate and/or blood pressure appeared to be used, not only for making and managing new diagnoses, but to guide prescribing decisions, namely drug and dose selection, in previously diagnosed hypertension (33/55), tachyarrhythmias (12/55), angina (7/55), or heart failure (3/55). In patients with hypertension, clinicians used patient-generated data to advise on current anti-hypertensive regimes; recommending regime maintenance (19/33), intensification (10/33), or de-prescribing (4/33).

### Impact of lockdown and comparison between traditional and novel monitoring devices

Between the start of 2020 and the start of the first lockdown (23^rd^ March 2020) in England there were 516 ICCS consultations with reference to self-monitoring in 43 instances (8.3%). In the 857 consultations post lockdown there were 142 mentions (16.6%). The difference was statistically significant (p < 0.001).

The mean age of those using emerging monitoring devices, namely smart watches, Fitbits™ or KardiaMobile™ was compared with patients using more traditional devices such as oscillometric blood pressure monitors and pulse oximeters. Those using traditional home monitoring devices were on average 67.2 (± 2.72) years old (range 29–89 years) and those using internet-connected devices 52.1 (± 4.68) years old (range 20–85 years). This 15-year difference between the two population means was statistically significant (p < 0.0001).

## Staff experiences and views

**Fig. 2:**
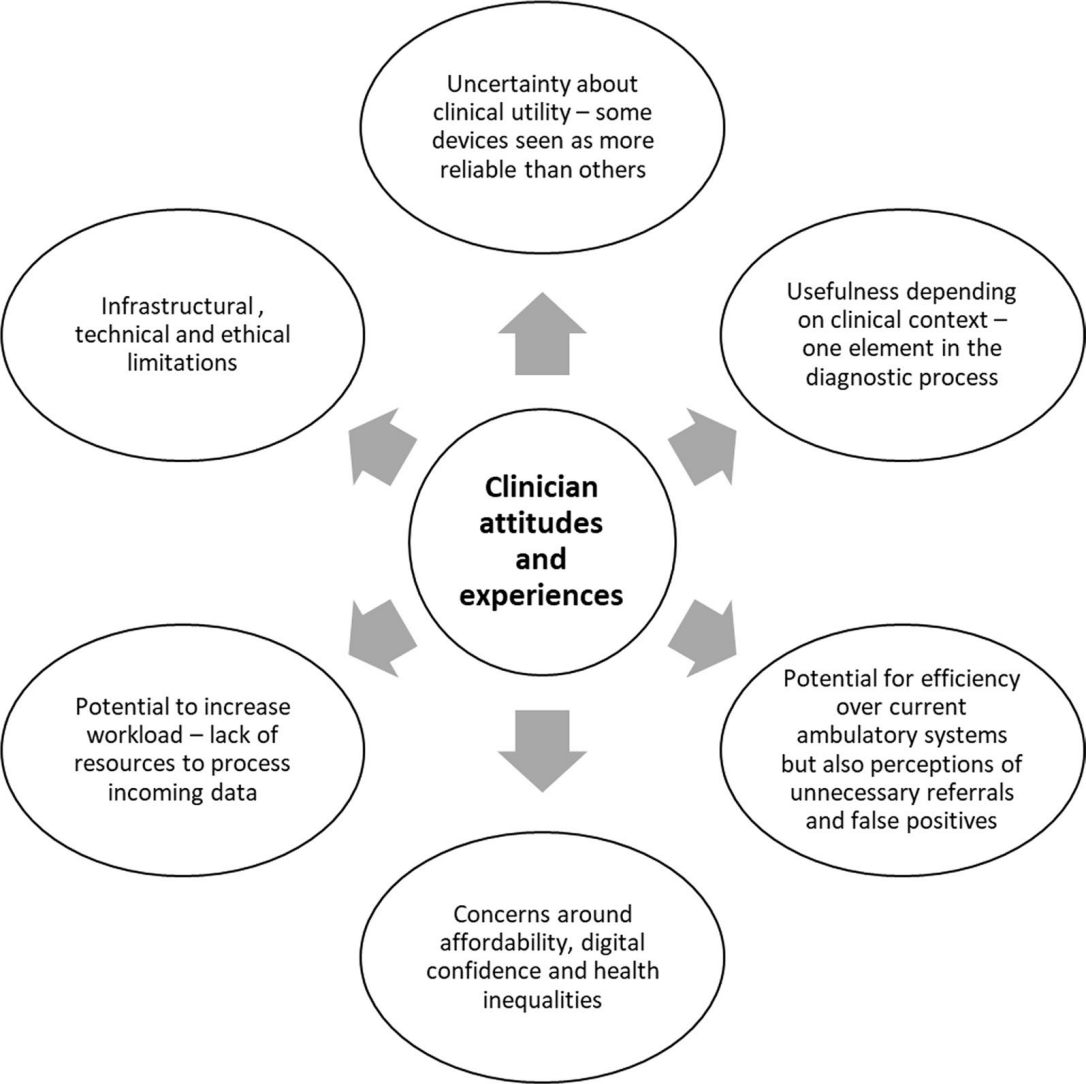
Summary of qualitative findings

Interviews with clinicians focussed on the contribution of patient-generated data to cardiac diagnoses, primarily of arrhythmias; and monitoring of long-term cardiac conditions, particularly heart failure. Most participants saw commercially available technology as having potential to improve care but highlighted technical, infrastructural and ethical limitations whilst also suggesting possible solutions (see Fig. 2 for a summary of findings).

### Potential diagnostic utility of self-monitoring data

Clinicians explained how self-monitoring devices could contribute useful data for some purposes but not others. They expressed doubts over using devices designed for recreational use for healthcare purposes and highlighted the finite value of pulse or heart rate monitoring without an accompanying ECG trace. Nonetheless, wearable activity trackers such as smart watches providing continuous heart rate monitoring were considered advantageous over handheld ECG recorders for capturing arrhythmias of short duration, associated with syncope, or triggered by exercise. For example, exercise-induced paroxysmal tachyarrhythmias have been detected serendipitously with continuous heart rate monitoring furnished by wearables or built into sports equipment.

‘[…] I’ve got dozens of examples […] you could see […] you know, from 90 to 290 beat per minute then a period of where it gradually slowed down as they stopped exercising and then a sudden termination. I’ve seen those in cycling and running. Those sorts of patterns are absolutely unmistakeable and they always reflect paroxysmal tachycardia. That’s one pattern of heart rate monitor that now is almost pathognomonic. Of course, you wouldn’t know the exact electrophysiological diagnosis because of not having the ECG, but it would prompt further investigation’ Arrhythmia sub-specialist, Interview 1

Devices providing single-lead ECG traces were viewed as ideal for capturing paroxysmal tachyarrhythmias, particularly those occurring infrequently.

‘I’ve had numerous patients who’ve picked up AF because of buying a Kardia device, where we otherwise might have struggled to diagnose it’. Arrhythmia sub-specialist, Interview 2

However, even with the NICE-endorsed device, diagnosing paroxysmal supraventricular tachycardias (pSVTs) was considered more difficult than diagnosing paroxysmal atrial fibrillation (PAF) because recording generally starts after symptom onset, missing the abrupt change in rate and P wave morphology helpful for a pSVT diagnosis. Yet in clinical contexts compatible with diagnosis of PSVT, and at rates exceeding most sinus tachycardias, clinicians reported making at least an umbrella diagnosis of pSVTs with this device. Even in pSVTs at slower rates overlapping with common sinus tachycardia (120-140 bpm), discernible altered p waves had enabled a diagnosis of pSVTs. Clinicians did not recall cases of higher risk tachyarrhythmias being diagnosed with patient-owned devices and it was suggested that in the presence of pointers to significant cardiac pathology, more established diagnostic approaches would be selected. In group discussion, clinicians highlighted that in a small number of cases, they were prepared to act on a patient’s single lead ECG device indicating a narrow complex tachyarrhythmia. In the absence of ‘red flag’ symptoms or adverse family history pointing to arrhythmogenic cardiomyopathy, and in the presence of a normal 12-lead ECG excluding pre-excitation, some clinicians felt comfortable using data supplied by patients to advise about self-termination techniques, trial rate limiting medications or refer for electrophysiological mapping and catheter ablation.

Automated oscillometric blood pressure monitors with irregular heartbeat indicators were viewed as unreliable for the detection of paroxysmal atrial fibrillation (AF), with uncertainty around which brands and models had secured independent validation of their accuracy, and recognition that despite built-in interpretative algorithms, frequent ectopy can trigger false alerts.

“But I don’t think I’d use it in a sort of de novo situation of someone who’s never known to have had an arrhythmia. I’d struggle to place any weight on it in that context.” [Arrhythmia sub-specialist, Interview 2]

A consultant reflected on how these devices’ predecessor, the mercury sphygmomanometer was, in the hands of an experienced clinician, highly effective for opportunistic detection of atrial fibrillation, a function lost with the move to mercury-free automation. Whether automated irregular heartbeat indicators can be optimised to restore the role of blood pressure monitors in opportunistic detection of paroxysmal atrial fibrillation remained an open question.

In discussing our quantitative data with participating clinicians, it was apparent how, even patient-generated data that might be considered diagnostically indeterminate, was treated by clinicians as a strand of evidence, woven into the diagnostic process. It was also apparent that whether data was eschewed or incorporated was greatly dependent on the clinical context. In cases where clinicians could not differentiate sinus tachycardia (related to exercise, deconditioning or anxiety) from a tachyarrhythmia, if the patient’s baseline risk was low, further investigations were kept to a minimum and the patient was reassured. Similarly, in cases where patients reported a slow pulse rate without accompanying ECG trace, its interpretation and impact was heavily influenced by context: younger patients, high vagal tone due to athleticism, diurnal variation, or symptoms typical of ectopy, tipped clinicians towards innocent diagnoses and reassurance, whereas in older patients or those with more worrying symptoms such as syncope, possible underlying conduction defects were actively sought by supplementary monitoring.

### Perceived advantages of self-monitoring over current diagnostic and monitoring systems

Multiple examples of home monitoring enabled diagnoses of AF, SVTs and exercise related paroxysmal tachyarrhythmias were contrasted with the inefficiency of current ambulatory diagnostic systems. Clinicians viewed self-monitoring as having the potential to reduce repeated hospital contacts, avoid unnecessary or unproductive investigations, cut costs and provide more convenience for patients.

Clinicians reflected on how, in patients with chronic heart failure, self-monitoring could contribute to reduction of unplanned hospitalisations through tighter, individualised management, accelerated optimisation of medical therapy, and enabling patients to undertake self-management with assumed restoration of personal agency.

‘….I can think of one particular patient who …… had severe heart failure and it was quite clear there was an optimal blood pressure for him which was round about a systolic pressure of about 70 or 80 which is quite common in heart failure populations for it to be relatively low. And he became very good at up-titrating and down-titrating his vasodilators according to blood pressure and his diuretics according to the weight……whereas previously, without self-monitoring, the patient had repeated admissions ‘3 times in 12 months either with dehydration or with overload’ Arrhythmia sub-specialist, Interview 1

### Challenges with patient self-monitoring: unnecessary referrals, anxiety and inequalities

The spectre of additional referrals resulting from self-monitoring was frequently raised by participants, who recalled referrals with exercise related sinus tachycardias detected by fitness monitors (in contradistinction to exercise related tachyarrhythmias). The rule of thumb guidance for a safe maximum heart rate of ‘220 minus age’ is one that may be used by fitness device manufacturers to trigger alerts. A consultant electrophysiology sub-specialist thought it inappropriate for such alerts to trigger a cardiological referral.

‘It’s an approximate guideline…. to be honest the only context in which I use it is to judge whether an exercise test has achieved an adequate workload. I’m not that interested in it otherwise because there’s so much variability in the heart rate. I’ve never really seen any convincing data to show if your heart rate goes to more than that it implies there’s necessarily anything wrong. It’s only that there’s so much variability in sinus rates.’ Arrhythmia sub-specialist, Interview 1

Also highlighted were referrals driven by both oscillometric blood pressure monitors and commercial heart rate monitors spuriously reporting a bradycardia in bigeminal or trigeminal rhythms, due to variable stroke volume. While these rhythms are not harmful to patients, apparent low heart rates can trigger alerts and ultimately an outpatient referral.

‘Another incredibly common source of over referral, I think one would probably see one referral a month you know because of people being bradycardic on various forms of pulse checks or sphygmomanometry and its down to bigeminal rhythms. Which again I don’t think is a type of referral you would have seen …. when I started as a consultant in 19... It does seem to be very much a phenomenon in the last 15–20 years.’ Arrhythmia sub-specialist, Interview 1

All clinicians highlighted how home monitoring can give rise to understandable concern, hypervigilance or anxiety in patients, and sometimes repeated, excessive blood pressure or heart rhythm monitoring. They recognised how arrhythmia alerts, with little explanation pending medical assessment, sometimes led to need for reassurance:

‘…..probably without any support from someone like Apple as to how to deal with that, you know you get it flagging up saying you’ve got an arrhythmia. Well, what do you about that? You might not be able to get an appointment to get an answer to find out whether it’s something dangerous or no’ Arrhythmia sub-specialist, Interview 2

Participants recognised that health inequalities could be accentuated by widespread adoption of consumer-led self-monitoring; patients unable to purchase equipment could suffer worse health outcomes or delayed diagnosis. Concerns about affordability made several clinicians hesitant about recommending patients buy a device, and others proposed means-testing to enable access. There were concerns around older patients with cognitive problems being unable to use digital solutions for self-monitoring, although some clinicians mentioned patients in older age groups becoming increasingly confident with technology in the Covid-19 context. Nonetheless, 14% (26/185) of the clinic letters mentioning self-monitoring related to clinician recommendations to purchase or use self-owned devices.

### Views on workload, infrastructure and risk management

There was a strong sense that self-monitoring introduced a new workload for clinicians that was ad hoc and not well integrated with usual care processes, either operationally, or culturally. Frequently raised was the need to review the data and feedback generating additional work outside the normal remit, uncosted and unresourced. The most widely cited organisational challenge to home monitoring across all interviews was the lack of infrastructure to deal with incoming information.

‘I think we all share some reservations about the way in which the data is gonna be fed back to us with the expectation of getting a report and the volume of data that we might get back’ Arrhythmia sub-specialist, Interview 2

Many conceded that the trend towards self-monitoring is ‘*inevitable’* and ‘*bound to grow’*, so it should be shaped by health professionals to ensure workload remains manageable, and appropriate clinician training, reimbursement and governance frameworks are in place (including clinician engagement with technology companies to shape developments in this area). Others were less convinced that patients’ self-monitoring data should be formally captured in patient records and care processes, and instead advocated for a more ‘*organic’* case-by-case approach based on patient need:

‘I would be slightly more circumspect about the assumption that we should be incorporating these into regular care, and that we need to set up systems for this. I think it will happen organically to some degree anyway, and we just need to have approaches that will allow their use where helpful. A small example: while setting up systems to capture the data into the EPR would be nice, in reality this is a huge piece of work and is not the top priority. Consultant cardiologist, Email discussion

There were concerns that diagnostic algorithms designed to minimise manufacturer culpability may lead to false positives, with the risk of alert fatigue, and subsequent errors, missed diagnoses or patient harm. Clinicians confessed to irritation in response to device-related referrals, such as those driven by diurnal rhythms, high vagal tone, ectopy or normal exercise related sinus tachycardia.

‘….. and you know there’s quite a lot of colleagues who …… just don’t want to know about it’ Arrhythmia sub-specialist, Interview 1

Clinicians were also looking for a more solid evidence base, either for heart failure monitoring, screening for atrial fibrillation or targeted case-finding, to be able to engage more purposefully with self-monitoring practices. Clinical and institutional inertia and lack of incentives to change the current system present barriers to adoption of patient self-monitoring data.

Drivers to adoption now include the Covid-19 pandemic related increase in remote consulting. Home monitoring has a close, although not exclusive interrelationship with remote consulting. The optimal level of remote consulting is perhaps yet to be determined but, pandemics apart, considerations included not only patient welfare and convenience, but also staff convenience and welfare, knock-on impacts on staff recruitment and retention, and wider still environmental impacts.

Participants discussed a range of solutions to manage an increasing workload from self-monitoring data and reduce avoidable referrals, such as triaging incoming referrals under certain criteria, better characterising criteria for referral from primary care, developing technology-driven intermediary triage processes that could be outsourced to technology providers (thus hoping to counteract national shortage of cardiac physiologists and specialist arrhythmia nurses), and more speculatively, automated data analysis using machine learning and artificial intelligence, although human interpretation was still considered important.

## Discussion

This mixed-methods observational study set out to quantify clinician recognition of self-monitoring and its influence on clinical decision-making in a community cardiology unit, including an understanding of staff views and experiences across the NHS Trust’s cardiology service. Over a 9-month period, patient self-monitoring activities were acknowledged in 13.5% community cardiology clinic letters. Albeit a small proportion, this was higher than anticipated at the time the service improvement project was conceived as there had been no specific endorsement of, or training around use of patient-initiated self-monitoring data. Mentions of self-monitoring doubled, from 8 to 16%, after the start of the first UK lockdown (March 2020). This may reflect an increase in patient self-monitoring and lifestyle changes driven by the pandemic. Equally, clinicians may have overcome recognised under-reporting of patient self-monitoring by patients [18] by actively enquiring and taking greater account of self-monitored data when, for a six-week period, face-to-face appointments were supplemented by telemedicine, and afterwards, when a mixed telemedicine and face-to-face service supervened.

Whilst the various devices recorded a range of physiological parameters, with some designed for sports and fitness tracking, patients and clinicians repurposed them to both evaluate symptoms and guide therapies. There was significant variation between clinicians mentioning self-monitoring in their letters, which could reflect different preferences in drawing on and documenting patient-initiated self-monitoring data. Diverse views were voiced in qualitative research and group discussions with some clinicians more willing to interpret and ‘trust’ self-monitoring data than others.

A tangible change that has emerged from the service improvement project is a more generalised understanding and readiness between ICCS clinicians to embrace data from devices providing downloadable single lead ECG traces. Clinicians learnt how these devices were often providing an immediate diagnosis of tachyarrhythmias. Consultant interviewees anticipated this finding whilst highlighting that in some contexts, particularly exercise induced tachyarrhythmias, heart rate alone can provide a useful lead. Device-stored ECG traces enabled firm diagnoses of atrial fibrillation more readily than the diagnosis and subclassification of paroxysmal supraventricular tachycardias (pSVTs), a limitation shared by traditional ambulatory monitoring techniques. There were no examples of high risk tachyarrhythmias amongst the 185 consultations examined in detail, perhaps reflecting their relative rarity in an outpatient setting as well as a disinclination expressed by clinicians to draw on unvalidated data sources in clinical contexts pointing to potentially lethal arrhythmias. The limited evidence concerning the accuracy of current commercially available devices for detection of ventricular arrhythmias has been highlighted [6, 19, 20].

Although the number of patients in whom narrow complex tachyarrhythmias (AF or SVTs) were diagnosed directly through self-monitoring was small (just 8 patients) it was notable that a high proportion (5) were referred on for non-pharmacological rhythm control interventions, all but one of whom were diagnosed with wearables. A high intervention rate might be explained by the fact that whilst prevalence of atrial fibrillation increases with age, symptom burden is often higher in younger groups; and the most common supraventricular tachycardia, atrioventricular nodal re-entrant tachycardia has a bimodal age distribution. The 5 patients accessing rhythm control therapies were younger than those managed by rate control. Interestingly, a similar finding, with an increased rate of catheter ablations for AF in patients using wearables compared with non-users has been reported [21]. This may indicate age-related differences in tolerability of tachyarrhythmias (as, like ours, this study also found the median age of wearables users was lower); increased patient awareness of the condition as a result of their wearables’ data; or that clinicians are nudged towards a rhythm control strategy by data from wearables. A need for prospective randomized long term evaluation of the association between wearables and healthcare usage and outcomes has been proposed [21].

In our quantitative study we found that prescribing decisions informed by self-monitoring data related not only to hypertension but to other previously diagnosed cardiovascular conditions. The benefits of self-monitored blood pressure data are increasingly well established, especially for management of hypertension [22, 23]. An online survey of 300 UK GPs in 2015 found 58% used self-monitored blood pressure for diagnosis and 84% for monitoring of hypertension [24]. Group discussions revealed that most, but not all of the extended role GPs staffing the ICCS service were routinely using home BP monitoring data for the management of hypertension in their primary care roles. Study data demonstrated they were also basing additional cardiovascular prescribing decisions, in tachyarrhythmias, angina and heart failure on recent self-monitored heart rate and blood pressure readings rather than one-off evaluations in primary care or the clinic. This pragmatic approach could be advantageous but to our knowledge lacks an evidence base. Device recorded heart rate (as opposed to blood pressure) is not mentioned as a useful element of telemedicine in trials of home blood pressure monitoring nor in recent literature advocating home monitoring and emphasising its importance during the pandemic [23, 25]. It should be acknowledged that for those patients consulted during lockdowns, patient self-monitoring was sometimes the only way physiological measurements within a relevant time frame could be obtained. Deployment of a similar approach has recently been described, with clinician instigated pre-appointment rate and rhythm monitoring being achieved with a specific mobile phone app to enable Covid-19 pandemic related telemedicine in patients with AF [26].

Previous research also supports our findings on the potential for widening health inequalities. Unsurprisingly, patients with AF using wearables identify as less socioeconomically deprived than propensity matched patients who are not using wearables [21]. Prices for validated home blood pressure monitors (HBPMs) are higher compared to HBPMs without evident validation, which also generates inequalities in the potential for generating ‘trustworthy’ self-monitoring data [27]. Device loans have been considered as a solution to affordability, with the 2021 NHS Plan in the UK promising greater funding to deprived areas and encouragement of digital innovation including distribution of BP monitors [1, 28, 29]. The challenges on equity due to increased use of digital technologies in healthcare (in what has been termed ‘techQuity’) have been further considered elsewhere [30, 31].

**Table:** 

**Box 1**. Implications for clinical care	
Commercially available self-monitoring technologies are in widespread use by patients and being incorporated into clinical care despite varying levels of clinician understanding, training, and trust and often without organisational endorsement or infrastructure. They bring potential for streamlined pathways but also unintended consequences including a small proportion of unwarranted referrals, significant when scaled up. Training, protocols and an overarching governance framework are needed to optimise the usage and value of self-monitoring in regular cardiovascular care. Targeted education for GPs and device users and research-based technological or software enhancements could facilitate the diagnosis of important rhythm disorders and mitigate unwanted outcomes	

Our study also highlights challenges with governance, infrastructure and resources to support incorporation of patient data into clinical decision-making, suggesting a potential disconnect between patient willingness to self-monitor, and organisational readiness to accommodate this. Our study adds further empirical data to a recent systematic review which found that the most frequent barriers to clinician uptake of digital technologies include the increased work related to the technology, concerns about reliability or limited evidence supporting its use, and lack of integration with electronic medical records [32]. Concerns of this sort can result in what has been described elsewhere as ‘a potentially corrosive sub-culture of pessimism about digital health.’[33]

Another commonly articulated concern was that patients worry about seemingly abnormal readings without clinical significance. The validity or at least proportionality of these concerns, frequently expressed by clinicians, has been questioned [4, 23, 24, 34]. In our study, just 6.5% (12/185) of letters could be characterised as representing ‘inappropriate’ referrals and arguably resulting to a ‘waste’ of healthcare resources. Although smaller in number than some clinicians assumed, scaled up over entire healthcare systems the volume of such referrals could be significant. Proactively addressing such ‘unintended’ consequences is an important part of evaluating or implementing digital health [33] and requires further examination in the context of technological innovations for cardiovascular conditions.

**Table:** 

**Box 2**. Suggestions for further research	
1	Improve understanding of accuracy and reliability of data generated by commercially available devices
2	Enhance differentiation between normal exercise-related sinus tachycardia and exercise-induced tachyarrhythmias; and between significant bradyarrhythmias and spurious bradycardia due to frequent ectopy
3	Identify impact on health inequalities from routine use of commercially available self-monitoring devices in cardiovascular care
4	Improve understanding of advantages and limitations of self-monitoring data compared to one-off clinical evaluation for cardiovascular prescribing decisions (e.g. in tachyarrhythmias, angina and heart failure)
5	Assess feasibility and cost-effectiveness of service models incorporating patient-generated cardiovascular data

## Limitations

Our qualitative study sample size was small, drawn from just one NHS Trust and all clinicians involved had cardiac training. This enabled in-depth, informed and insightful discussion of issues surrounding self-monitoring but may not be generalisable to the wide range of healthcare professionals with whom patients may share data.

Our pragmatic study provides a snapshot of how often clinicians find self-monitoring data worthy of inclusion in their letters, either because it helped clinical evaluation or to demonstrably address patient concerns. Yet, it is likely that not all instances of self-monitoring find their way into clinic letters. Patients may self-monitor without reporting to their clinician; equally clinicians may neglect to mention instances of monitoring if they deem it insignificant, unhelpful or if they distrust the device data, leading to underestimation of the prevalence of patient-initiated self-monitoring. Clinicians may also introduce bias by reporting patient-generated data more often when it contributes to diagnosis or management, skewing estimations of potential utility.

## Conclusions

Detailed analysis of consultation letters and qualitative interviews provided insights into how cardiologically-trained GPs drew on patient self-monitoring data for clinical decision-making, including during the first Covid-19 lockdown in England. Data indicate limited reliance on remote monitoring overall but increased usefulness in maintaining care provision during the pandemic. Clinicians revealed a complex and nuanced relationship with patient-initiated self-monitoring. They saw a place for both digital and traditional devices in selected situations, recognising instances of accelerated diagnoses, greater convenience and patient enablement, but also potential for aggravation of health inequalities. There were co-existing concerns about reliability, workload and governance, patient anxiety and escalation of unnecessary referrals.

Although enthusiasm for integration of patient-initiated self-monitoring data in clinical decision-making varied considerably amongst clinicians, overall use as reported in clinical letters was greater than anticipated. Increased remote monitoring exploiting both implanted and patient-owned devices in the context of the Covid-19 pandemic appears to have shifted thinking in many clinicians, despite residual uncertainties.

## Data Availability

Quantitative and qualitative datasets available on request.

## Abbreviations

AVNRT: Atrio-ventricular nodal re-entrant tachycardia
SVT: Supraventricular tachycardia
AF: Atrial fibrillation
PAF: Paroxysmal atrial fibrillation
BP: Blood Pressure
FDA: Food and Drugs Administration
NICE: National Institute for Care and Excellence
NHS: National Health Service
ICCS: Integrated Community Cardiology Service
ECG: Electrocardiogram
GP: General practitioner
